# Deep-Learning-Based Real-Time and Automatic Target-to-Background Ratio Calculation in Fluorescence Endoscopy for Cancer Detection and Localization

**DOI:** 10.3390/diagnostics12092031

**Published:** 2022-08-23

**Authors:** Yang Jiang, Jing Chen, Chen Gong, Thomas D. Wang, Eric J. Seibel

**Affiliations:** 1 Department of Mechanical Engineering, University of Washington, Seattle, WA 98195, USA; 2 Division of Gastroenterology, Department of Internal Medicine, University of Michigan, Ann Arbor, MI 48109, USA; 3 Department of Mechanical Engineering, University of Michigan, Ann Arbor, MI 48109, USA; 4 Department of Biomedical Engineering, University of Michigan, Ann Arbor, MI 48109, USA

**Keywords:** cancer detection, CAD, deep learning, real time, segmentation, multimodal fluorescence endoscopy

## Abstract

Esophageal adenocarcinoma (EAC) is a deadly cancer that is rising rapidly in incidence. The early detection of EAC with curative intervention greatly improves the prognoses of patients. A scanning fiber endoscope (SFE) using fluorescence-labeled peptides that bind rapidly to epidermal growth factor receptors showed a promising performance for early EAC detection. Target-to-background (T/B) ratios were calculated to quantify the fluorescence images for neoplasia lesion classification. This T/B calculation is generally based on lesion segmentation with the Chan–Vese algorithm, which may require hyperparameter adjustment when segmenting frames with different brightness and contrasts, which impedes automation to real-time video. Deep learning models are more robust to these changes, while accurate pixel-level segmentation ground truth is challenging to establish in the medical field. Since within our dataset the ground truth contained only a frame-level diagnosis, we proposed a computer-aided diagnosis (CAD) system to calculate the T/B ratio in real time. A two-step process using convolutional neural networks (CNNs) was developed to achieve automatic suspicious frame selection and lesion segmentation for T/B calculation. In the segmentation model training for Step 2, the lesion labels were generated with a manually tuned Chan–Vese algorithm using the labeled and predicted suspicious frames from Step 1. In Step 1, we designed and trained deep CNNs to select suspicious frames using a diverse and representative set of 3427 SFE images collected from 25 patient videos from two clinical trials. We tested the models on 1039 images from 10 different SFE patient videos and achieved a sensitivity of 96.4%, a specificity of 96.6%, a precision of 95.5%, and an area under the receiver operating characteristic curve of 0.989. In Step 2, 1006 frames containing suspicious lesions were used for training for fluorescence target segmentation. The segmentation models were tested on two clinical datasets with 100 SFE frames each and achieved mean intersection-over-union values of 0.89 and 0.88, respectively. The T/B ratio calculations based on our segmentation results were similar to the manually tuned Chan–Vese algorithm, which were 1.71 ± 0.22 and 1.72 ± 0.28, respectively, with a *p*-value of 0.872. With the graphic processing unit (GPU), the proposed two-step CAD system achieved 50 fps for frame selection and 15 fps for segmentation and T/B calculation, which showed that the frame rejection in Step 1 improved the diagnostic efficiency. This CAD system with T/B ratio as the real-time indicator is designed to guide biopsies and surgeries and to serve as a reliable second observer to localize and outline suspicious lesions highlighted by fluorescence probes topically applied in organs where cancer originates in the epithelia.

## 1. Introduction

Esophageal cancer is the eighth most common cancer in the world. With a poor 5-year survival rate of less than 20%, this cancer ranks sixth in mortality worldwide [1]. Esophageal adenocarcinoma (EAC), the prevalent subtype of esophageal cancer in developed countries, is rising in incidence due to increased risk factors such as gastroesophageal reflux disease and obesity [2,3]. Barrett’s esophagus (BE), a known precursor for EAC, transforms via low-grade dysplasia (LGD) and high-grade dysplasia (HGD) before progressing to EAC [4]. Due to the lack of an efficient early detection method for Barrett’s neoplasia, EAC is usually diagnosed at an advanced stage with poor patient prognosis.

Standard endoscopy screening with white light illumination and random, four-quadrant biopsy is recommended for cancer surveillance in BE patients but is labor-intensive and limited by sampling error [5]. Novel endoscopic imaging technologies with molecular agents targeting cancer-specific biomarkers have been developed for early EAC detection [6]. In our previous studies, a scanning fiber endoscope (SFE) with fluorescence-labeled peptides has demonstrated feasibility to visualize the expressions of EGFR and ErbB2 for Barrett’s neoplasia detection [7,8]. To quantify the fluorescence images, target-to-background (T/B) ratios were calculated. These T/B ratios measured the ratios between the average intensities of high-contrast fluorescence targets and the neighboring background regions [7]. This quantitative ratio can be more important for more widespread use of fluorescence molecular probes during cancer surveillance and screening without an expert endoscopist. In this case, a computer-aided diagnosis (CAD) system with real-time and automatic T/B ratio calculation can serve as a reliable trained observer to localize and outline suspicious lesions for biopsy guidance and cancer detection. This real-time T/B calculation is limited by the speed of target segmentation. Currently, the Chan–Vese algorithm is applied to segment the fluorescence targets, with speed limited to only 2–3 frames/s [9]. Since the Chan–Vese approach evolves the level set iteratively to minimize the energy function, the algorithm is not easily computed in parallel processing, which impedes real-time computation. In addition, hyperparameters need to be adjusted for in the Chan–Vese algorithm when the contrast and brightness of the images change, which makes automatic T/B calculation more difficult. Moreover, to achieve this automatic CAD system, an automatic frame selection is needed to reject frames with diverse image artifacts, including (1) saturation, (2) dye pooling, (3) air bubbles, and (4) instruments (mother endoscope), as well as to select frames with suspicious lesions for further segmentation and T/B quantification, which has been performed manually in our previous studies [7,8].

Deep learning, a machine-learning subfield, has been successfully applied to many areas of science and technology, including computer vision [10,11], speech recognition [12,13], games [14], and bioinformatics [15], and has been expanded to the field of medical image analysis. Recently, there have been significant developments in computer-aided detection and diagnosis using deep-learning techniques in various medical-imaging modalities, e.g., MRI, computed tomography, and ultrasounds [16,17]. The main contribution of deep learning in medical image analysis is mainly around two topics: (1) detection and classification, and (2) localization and segmentation.

### 1.1. Detection and Classification

Convolutional neural networks (CNNs) have become a state-of-the-art tool for medical image classification and have been widely used for cancer classification. Litjens et al. used a CNN to detect cancer areas from H-and-E-stained whole slides for prostate and breast cancer detection. All the slides containing prostate cancer and metastases of breast cancer could be identified automatically, while 30–40% of the slides containing benign and normal tissue could be excluded without any human intervention [18]. Ardila et al. proposed end-to-end lung cancer screening with three-dimensional deep learning on low-dose chest computed tomography and achieved a state-of-the-art performance, with a 94.4% area under the curve (AUC) on 6716 National Lung Cancer Screening Trial cases [19]. Kooi et al. showed that a CNN model trained on a large dataset of mammographic lesions (45,000 images) outperformed a state-of-the art CAD system trained using manually designed features (respective AUC: 92.9% vs. 90.6%) [20].

#### Localization and Segmentation

Another contribution of deep learning to the medical-imaging field is the potential to improve the speed and accuracy of cancer localization and segmentation in clinical settings. A CNN-based algorithm was applied for the automatic localization and segmentation of rectal cancers on multiparametric MR imaging, with segmentation results comparable to those manually labeled by experts and a Dice similarity of 0.70 [21]. A hybrid network that fused DenseNet and UNet [22] together achieved a competitive performance for liver and tumor segmentation from 3D CT volumes. Features from both inter- and intraslices were extracted and jointed together for better 3D segmentation [23]. A two-pathway CNN that extracted both local and global contexts was applied to segment brain tumors from MRI scans and showed improved accuracy and speed over other algorithms reported on the 2013 BRATS [24].

There has also been an increasing number of studies that have applied deep learning to advance endoscopic imaging [25,26,27,28]. With the development of massive parallel architecture, or graphic processing units (GPUs), deep-learning techniques have demonstrated promising results in real-time endoscopic cancer surveillance [25,26]. Wang et al. developed a deep-learning algorithm by training it with 5545 colonoscopy images from 1290 patients for polyp detection and localization, with a processing speed of 25 frames per second. The algorithm achieved 94.38% sensitivity and 95.92% specificity validated on 27,113 colonoscopy images [25]. Although the segmentation of fluorescence images remains challenging due to the “patchy” appearance of the fluorescence targets, nonuniform intensities in both targets and backgrounds, and losses in contrast and resolution due to the scattering of light from biological tissue, there have been deep-learning models showing promising results for nucleus segmentation in fluorescence microscopy [29,30]. The robustness and real-time capability of deep learning allow real-time and automatic T/B calculation, while the training of deep-learning models generally requires a dataset with pixel-level lesion labels as ground truth [29].

In this paper, we present the development and validation of our deep-learning-based pipeline for automatic and real-time T/B calculation with frame-level diagnosis as ground truth only, which can be embedded in a CAD system for cancer surveillance and screening using fluorescence endoscopy with molecular agents. The proposed pipeline has two steps, with real-time and automatic suspicious frame selection in Step 1 and segmentation and T/B quantification in Step 2. The use of the T/B ratio preserves the clinically validated quantitative neoplastic lesion analysis for classification, unlike pure “black box” approaches to cancer detection and classification [31]. In Step 1, the frame-based diagnostic ground truth is used for training, and predicted suspicious frames in test videos and originally labelled frames can be combined as a larger segmentation dataset for Step 2. The labels of the segmentation training set are generated by a manually tuned Chan–Vese algorithm. The labor is reduced for labelling more nondiagnostic frames by looking into the predicted suspicious frames only.

The speed of Step 1 in our pipeline reaches 50 fps, and Step 2 is 15 fps. The efficient frame selection in Step 1 further improves the real-time capability of the T/B ratio diagnosis compared with the segmentation models in Step 2 only. To the best of our knowledge, there is no deep-learning-based CAD system for wide-field fluorescence endoscopy to provide (1) real-time suspicious frame selection or (2) real-time and automatic quantification using T/B ratios. In the experiment, we validate each step of the system in two clinical studies using fluorescence-labeled peptides for Barrett’s neoplasia detection.

## 2. Materials and Methods

**Figure 1:**
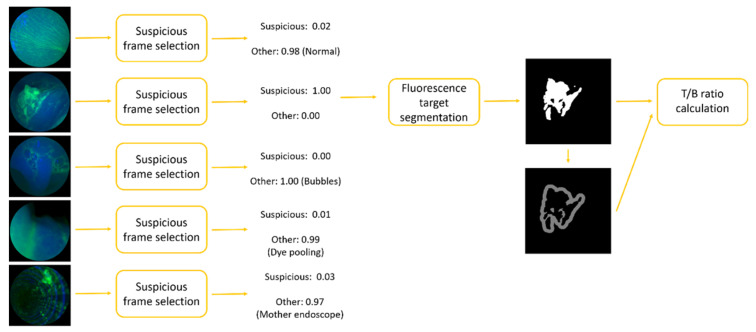
Overall CAD pipeline for automatic and real-time T/B calculation with two steps: (1) suspicious frame selection model and (2) fluorescence target segmentation model.

The whole CAD system could be divided into steps: (1) a suspicious frame selection model and (2) fluorescence target segmentation. The segmented targets were then used to generate a 30-pixel-wide background via morphological operations. T/B ratios were calculated from the mean intensities of each region. The pipeline is shown in Figure 1.

We trained different CNN architectures as backbones to extract features for the following tasks: (1) classification and (2) segmentation. These CNN architectures usually consisted of the following layers: convolutional layers, batch normalization layers, activation layers, pooling layers, and skip connection layers. All these layers were assembled to form different backbone architectures for feature extraction. In this paper, two different backbone architectures, MobileNetV2 [32] and Xception [33], were used.

### 2.1. Suspicious Frame Selection Model

**Figure 2:**
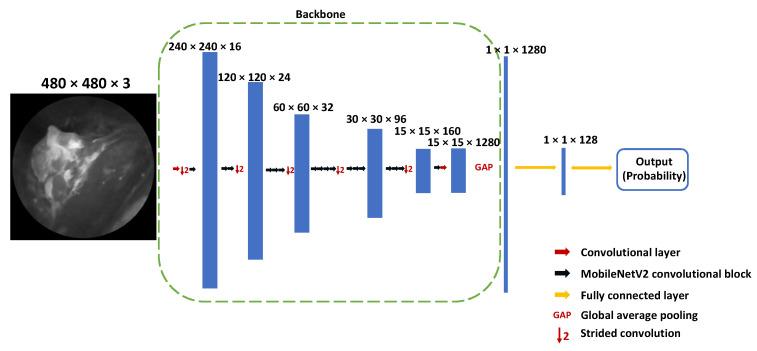
Suspicious frame selection model architecture with MobileNetV2 backbone.

Network architectures with Xception or MobileNetV2 backbones, followed by a global average pooling layer and 2 fully connected layers, were applied for suspicious frame selection. The whole architecture for frame selection is shown in Figure 2.

### 2.2. Fluorescence Target Segmentation Model

Architectures for semantic segmentation usually consist of two parts: an encoder and a decoder. The encoder compresses the input image into smaller vectors with context information. On the other hand, the decoder expands the extracted information and reconstructs an output with the size of the original input image to obtain a pixel-level classification. In this study, 2 different architectures, UNet and BiSeNet, were evaluated for fluorescence target segmentation.

**Figure 3:**
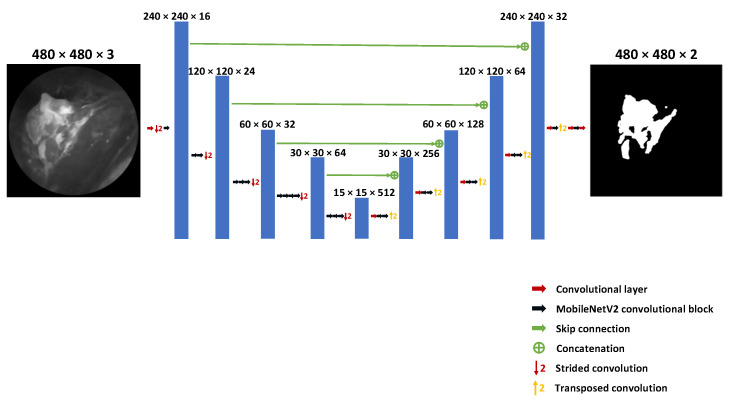
UNet architecture with MobileNetV2 backbone for fluorescence target segmentation.

UNet [22] consists of a symmetric encoder and decoder. The encoder extracts feature vectors from the input images. The decoder combines spatial information from different levels with up-convoluted context information. In this study, Xception and MobileNetV2 were used as backbones to extract features for the encoder. Figure 3 shows an example of the UNet architecture with the MobileNetV2 backbone.

**Figure 4:**
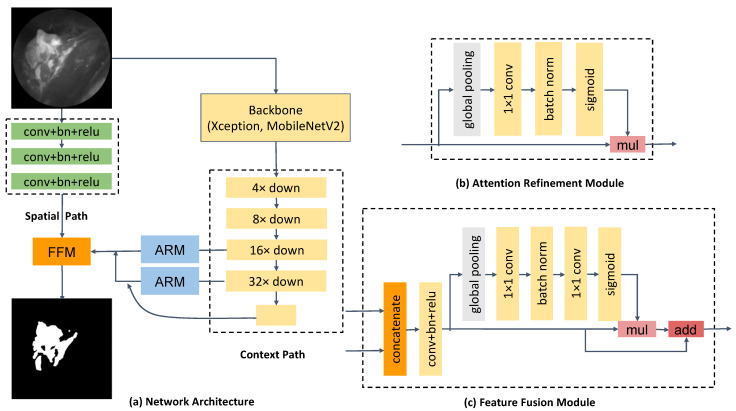
An overview of the bilateral segmentation network consisting of (**a**) network architecture, (**b**) components of the attention refinement module (ARM), and (**c**) components of the feature fusion module (FFM) [34].

BiSeNet33 contains two pathways, a spatial path with a small stride to preserve the spatial information and to generate high-resolution features and a context path as the encoder to obtain a sufficient receptive field and to extract context information for segmentation. Xception and MobileNetV2 were used as backbones to extract features within the context path. An attention refinement module was introduced to refine the features of the last two stages of the context path. A feature fusion module was introduced to combine features from the two pathways efficiently. The overall architecture of BiSeNet is shown in Figure 4.

### 2.3. Regularization

Different training techniques were applied to decrease overfitting and to improve the model performance without sacrificing the training cost. In this study, data augmentation was applied to increase the variability of the training images. We used 3 different types of image transformations for augmentation: (1) geometric distortion, (2) brightness and contrast adjustment, and (3) image blur and noise. For geometric distortion, we used random scaling, shifting, flipping, and rotating in the full range of 0–90 degrees. For blur and noise, we used random motion blur, Gaussian blur, and Gaussian noise. All the augmentation methods mentioned above were pixel-wise adjustments, which retained the original pixel information. “Early stopping” was applied to avoid overfitting [35]. Models with the maximum validation accuracy were saved. In addition, we used weights that were pretrained on the ImageNet data corpus as initialization for our backbone architectures. Natural images in the ImageNet dataset share fundamental features that can be transferred to medical images [36].

## 3. Experiments

### 3.1. Datasets and Preprocessing

Two datasets from two clinical studies were used for training and testing the deep-learning models presented in this work:

- The first set contained a total of 15 videos from an in vivo Multiplex imaging study (NCT03589443) performed in patients with Barrett’s neoplasia. The fluorescence-labeled peptides of QRH*-Cy5 and KSP*-IRDye800 targeting EGFR and ErbB2, respectively, were topically applied in the lower esophagus. Videos were recorded using SFE with a resolution of 720 × 720 at 30 Hz. Separate channels were used to record fluorescence images from QRH*-Cy5 (red) and KSP*-IRDye800 (green), as well as reflectance (blue). The combined duration of 15 videos was approximately 96 min (~173,000 frames) [7].
- A separate set contained a total of 20 videos from an in vivo Dimer imaging study (NCT03852576) performed in patients with Barrett’s neoplasia. Only IRDye800 was used to label dimer peptide QRH*-KSP*-E3-IRDye800, targeting both EGFR and ErbB2, and was again topically applied. The NIR fluorescence projected to the green channel was recorded with reflectance as the blue channel. The combined duration of 20 videos was approximately 88 min (~158,000 frames) [8].

All the images were preprocessed to have the same format before being passed to the deep-learning models. First, the individual frames were rescaled to a fixed size of 480 × 480 pixels to reduce the number of operations and to increase the model processing speeds. Then, the values of the pixels in each frame were normalized by dividing by 255. For the suspicious region detection task, fluorescence was projected to the green channel with reflectance as the blue channel. Each image from the Multiplex dataset was converted to two separate images. For the fluorescence target segmentation task, the fluorescence channels were extracted and converted to greyscale.

### 3.2. Model Development

We trained and evaluated the suspicious frame selection model and the fluorescence target segmentation model separately.

#### 3.2.1. Frame Selection Model

The preprocessed images were passed to the frame selection model and were classified into two classes: (1) frames containing bright, suspicious lesions and (2) frames containing a normal esophagus or frames with different artifacts, including dye pooling, air bubbles, saturation, or an instrument (mother endoscope). The datasets from the two clinical studies were divided into the following categories. 

Training and validation: 3427 images were extracted from 15 videos of the Multiplex study and 10 videos from the Dimer study. There were 1475 images with suspicious bright regions and 1952 images with different artifacts. The 3427 images were then split into training (83%) and validation (17%) groups under random and stratified sampling.

Testing: 1039 images were extracted from the remaining 10 unused videos from the Dimer study, which contained 443 images with suspicious bright regions and 596 images with artifacts.

We augmented the dataset by using different combinations of image transformations during training. We adjusted the batch size and optimized the initial learning rate for each backbone architecture individually. We employed the same learning rate schedule (decreasing by 0.8 every 10 epochs) for each backbone. The networks were trained with the ADAM optimizer [37] to minimize the binary cross-entropy loss.

In terms of evaluation, area under the curve (AUC) values of the ROC curve were computed and compared for different combinations of backbone and augmentation techniques over the test set, which measured the model performances irrespective of the chosen classification threshold. The accuracy, sensitivity, specificity, and precision were calculated for the model with the highest AUC. A gradient-weighted class activation map (grad-CAM) was applied to visualize the implicit attention of the trained CNN model. The discriminative features of the objects were highlighted by the grad-CAM, which provided a direct interpretation of the network when making its classification [38].

#### 3.2.2. Fluorescence Target Segmentation Model

Different model architectures of BiSeNet or UNet with backbones of MobileNetV2 or Xception were trained and evaluated for fluorescence target segmentation. Only frames with suspicious regions were selected from the two clinical studies for the model training and evaluation.

- Training and validation: 1006 images from 11 videos from the Multiplex study were randomly separated into training (910) and validation (96) groups;
- Testing A: 100 images were extracted from 4 different videos from the Multiplex study;
- Testing B: 100 images were extracted from the Dimer study.

All the images were paired with a pixel-level annotated segmentation map, which was used as ground truth for segmentation. The annotated segmentation maps were generated using the Chan–Vese algorithm. We applied the same training protocol for training the frame selection model. Different combinations of image augmentation were evaluated. The batch size and optimized initial learning rate were adjusted for each model and backbone individually. The ADAM optimizer was used to minimize the sparse, categorical cross-entropy. The performance was measured in terms of accuracy and pixel intersection-over-union (IOU) and averaged across the 2 classes [39]:

(1) $$IOU\left(A,B\right)=\frac{A\cap B}{A\cup B},$$

where A is the ground truth and B is the segmented fluorescence target.

Herein, we used a Windows computer with an Intel(R) Core(TM) i7-1065G7 CPU, a Toshiba NVMe KIOXIA 512 GB SSD, and an NVIDIA RTX 2080 SUPER GPU with a CUDA 10.0 for training and evaluation. All the experiments were implemented using Tensorflow [40] software library, an open-source deep-learning library.

## 4. Results

### 4.1. Suspicious Frame Selection

The suspicious frame selection results are shown in Table 1. Networks initialized from weights pretrained on the ImageNet data corpus demonstrated better performances compared to those starting from random weights. Different combinations of image augmentation techniques were evaluated. Networks with geometric distortions surpassed those without geometric distortions by a significant margin (one-tailed paired *t*-test, *p*-value). Neither (1) adjusting the brightness and contrast of the image nor (2) adding noise or blur to the image during training showed an improvement in model performance. Networks using the Xception backbone performed slightly better than those with the MobileNetV2 backbone (one-tailed paired *t*-test, *p*-value of 0.012).

**Figure 5:**
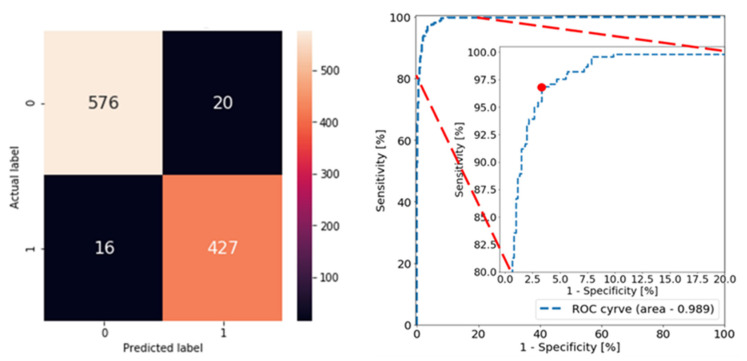
The confusion matrix and ROC curve for the best model (Xception backbone with geometric distortion, brightness, and contrast adjustment augmentation during training) evaluated over test data.

At a sensitivity level (true positive rate) of 96.4%, the best model using the Xception backbone with geometric distortion, brightness, and contrast adjustment augmentation during training (highlighted in bold in Table 1) had a specificity of 96.6%, a precision of 95.5%, and an overall accuracy of 96.8%. The confusion matrix and the ROC of the test data are shown in Figure 5. As can be seen, there was a reasonably large range of high sensitivities, high specificities, and high precisions (all greater than 90%).

**Figure 6:**
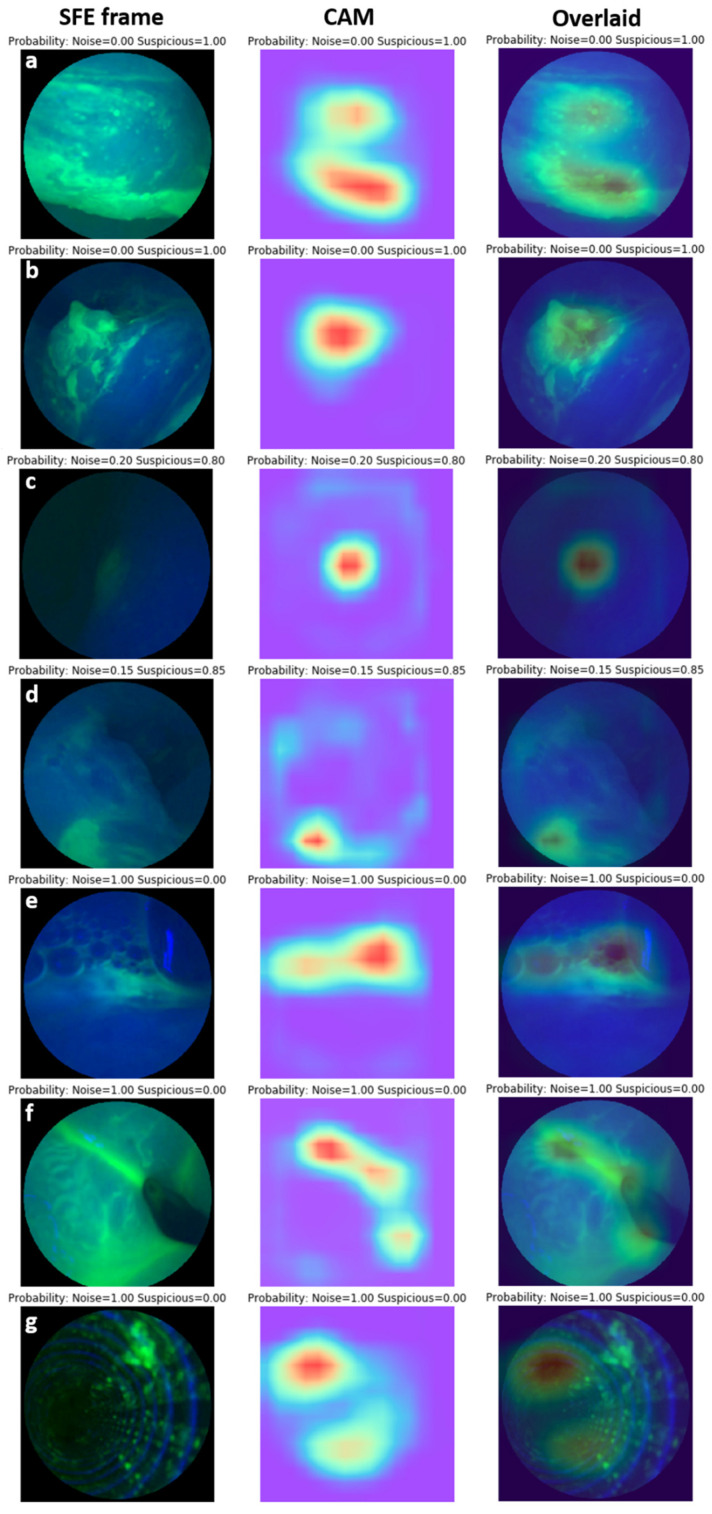
Examples of suspicious frame selection model visualized using grad-CAM. (**a**–**d**) Lesions from different locations with different brightness and contrasts were detected and used as features for classification. The heatmaps generated by grad-CAM overlaid well with the lesions. (**e**) Air bubbles were detected as artifacts and highlighted by grad-CAM. (**f**) Dye pooling in gastroesophageal (GE) junction was detected and classified as noise. (**g**) Image of the inside of the mother endoscope working channel was classified as artifact frame in spite of the debris in the top left corner appearing as a suspicious lesion. The model used features from other regions (highlighted by grad-CAM) for classification.

A grad-CAM was applied to visualize the discriminative features detected by the network. As can be seen in Figure 6a–d, fluorescence-highlighted lesions from different locations with different brightness and contrasts were detected and used as features for classification. Figure 6e–g demonstrated that the trained model correctly distinguished artifacts such as air bubbles, pooled dye, and the mother endoscope and classified these frames as noise.

### 4.2. Fluorescence Target Segmentation

A total of 1006 images were used to train and validate the model for the fluorescence target segmentation task. The test set results, as presented in Table 2 and Table 3, showed the following:

(1) Networks with geometric augmentation during training surpassed those without geometric augmentation by a significant margin (one-tailed paired *t*-test, *p*-value), which was consistent with our findings for the frame selection model;
(2) Neither (a) adjusting the brightness and contrast of the images nor (b) adding noise or blur to the images during training showed an improvement in performance;
(3) UNet outperformed BiSeNet in segmentation accuracy (one-tailed paired *t*-test, *p*-value);
(4) Using pretrained ImageNet weights as initialization did not improve the performance;
(5) BiSeNet models using the Xception backbone outperformed BiSeNet models using the MobileNetV2 backbone regarding to accuracy and mIOU (one-tailed paired *t*-test, *p*-value).

**Figure 7:**
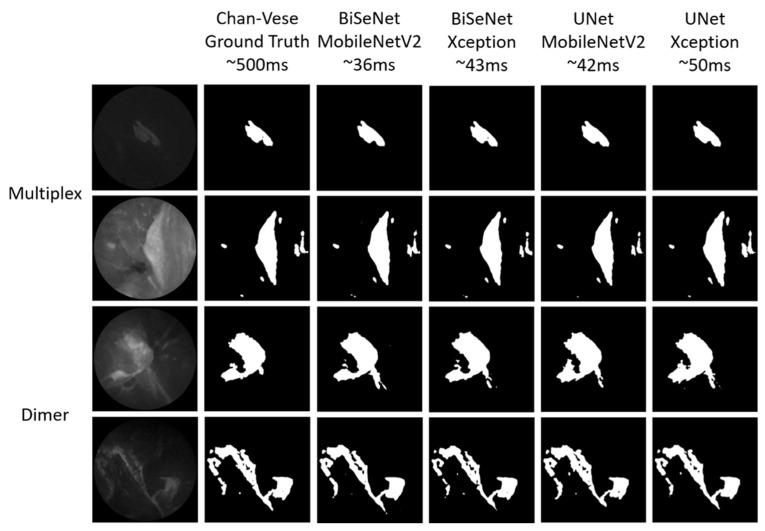
Representatives of segmentation results for BiSeNet and UNet models with MobileNetV2 and Xception backbones.

The UNet model with the MobileNetV2 backbone, geometric distortions, and image blur and noise augmentation performed better when segmenting the fluorescence images (highlighted in red in Table 3) and achieved mIOU values of 0.89 and 0.88 on the Multiplex and Dimer test datasets, respectively. Figure 7 shows examples of fluorescence target segmentation results tested on the Multiplex and Dimer datasets.

To better evaluate the accuracy of the whole pipeline for T/B calculation, we compared the T/B ratios calculated using the deep-learning pipeline with those using the Chan–Vese algorithm. A total of 58 frames with unique, suspicious bright regions were selected from 31 patient videos from the Dimer study. The average T/B ratios from the segmentation results of our best model for deep learning and the Chan–Vese algorithm were 1.71 ± 0.22 and 1.72 ± 0.28, respectively, with a *p*-value of 0.872 calculated using a two-tailed paired *t*-test. There was no significant difference when comparing the deep-learning T/B ratios with the Chan–Vese T/B ratios, which meant our segmentation deep-learning model could reach the accuracy of the labels we used for training.

### 4.3. Speed

We achieved average frame-processing times of 20 ms for the frame selection task and 42 ms (UNet with MobileNetV2) for the segmentation task with an NVIDIA RTX 2080S external GPU. In comparison to the ground truth Chen–Vese method, all the deep-learning methods increased in speed by ≥10×. The total processing time of the CAD system for each frame, including frame selection, fluorescence target segmentation, and T/B calculation, was around 70 ms.

## 5. Discussion

In this study, a deep-learning CAD pipeline was developed to achieve real-time and automatic T/B calculation. This research was an extension of our prior work, where we have shown that the T/B ratio can be used as a quantitative and accurate indicator for Barrett’s neoplasia detection and localization [7,8]. When combined with fluorescence endoscopy (SFE) and fluorescence molecular probes, this deep-learning CAD pipeline could serve as a reliable observer to localize and outline suspicious lesions for guiding biopsies, which has the potential to greatly improve routine surveillance for BE patients. Our study highlighted the development of artificial intelligence in fluorescence endoscopy undergoing clinical trials, specifically SFE for early EAC detection in a limited subject population at a tertiary referral hospital.

The reported pipeline consisted of two deep-learning models: (1) suspicious frame selection and (2) fluorescence target segmentation. Frames with suspicious bright regions were automatically selected, while frames with normal squamous cells or artifacts, including dye pooling, air bubbles, saturation, etc., were rejected in the frame selection model. A CNN model with an Xception backbone for frame selection achieved a uniform and consistent high performance with a sensitivity of 96.4%, a specificity of 96.6%, a precision of 95.5%, an overall accuracy 96.8%, and an AUC of 0.989 (Table 1). This suggests that the model likely offered sufficient sensitivity for selecting suspicious frames for further T/B calculation. To better unmask the “black box” of deep learning and to better understand how the models made their decisions, a grad-CAM was applied. The features highlighted by the grad-CAMs showed consistency with those selected by experienced human observers. As can be seen in Figure 6e,f, frames with air bubbles and dye pooling in the GE junction were rejected for further quantification since these features could diminish the robustness of the T/B ratio in esophageal neoplasia detection. On the contrary, frames with patchy lesions lightened by fluorescence probes were selected. The models learned these features for classification by themselves.

In an attempt to segment the fluorescence target, UNet and BiSeNet architectures with Xception and MobileNetV2 backbones were evaluated on two different datasets from two clinical studies. Different combinations of augmentation methods were tested to improve the model performance. As shown in Table 3, the best model, UNet with the MobileNetV2 backbone with added geometric distortions and image blur augmentation during training, achieved mIOU values of 0.89 and 0.88, respectively, for the Multiplex and Dimer studies. The T/B ratio calculated from the segmentation results had 1% difference compared with that calculated from the manually tuned Chan–Vese method, which generated training labels for our segmentation learning. The segmentation models trained only using images from one clinical study showed comparable performances to the images from the other clinical study. This finding demonstrated that the models could be generally applied to studies using SFE with different molecular probes for cancer detection in the esophagus, and most likely in the oral cavity [41], bladder [42], and colon [43,44], where topically applied fluorescence probes demonstrate early detection in the epithelia.

In our training dataset, we only had frame-based diagnostic labels without pixel-level lesion masks from pathologists. To leverage the classification labels thoroughly and embed them into the loss function, the suspicious frame selection model was separated. In the supervised training of the segmentation model, the labels were generated with a manually fine-tuned Chan–Vese algorithm for different frames. Continued improvements would be fusing the two-step model into one model for both Barrett’s neoplasia detection and localization when segmentation labels from pathologists are available. In this case, the segmentation models can be trained directly on the video frames. Architectures such as Mask R-CNN [45] and YOLACT [46] could be applied to localize each object in the image, including EAC and HGD lesions, dye pooling, and forceps. Instance segmentation masks could also be generated for each neoplasia lesion for T/B calculation. However, this task would require a relatively large volume of patient data to train the models, which could eventually be fulfilled when the technique is utilized in a multisite clinical trial for BE surveillance. Obtaining the pixel-level object masks as data annotations is labor-intensive, and label noise is a common feature of medical image datasets. The major sources of label noise include interobserver variability, human annotator error, and errors in computer-generated labels [47,48]. In addition to changing the network architectures for noise-labeling specifically [49,50], a large number of studies have kept the original network and only modified the loss functions [51,52], learning to reweight training data based on their noise levels [53], exploit data and label consistency [54], and optimize training procedures [55,56]. In a future study with obtained segmentation labels, further explorations should be conducted to train with noisy labels.

Regarding the speed of the deep-learning models, we achieved averages of ~20 ms for suspicious frame selection and ~42 ms for fluorescence target segmentation on images of 480 × 480 pixels using a single-threaded PC with an NVIDIA RTX 2080S external GPU. This is comparable to studies where deep learning has been investigated with endoscopic imaging for real-time cancer detection. By using a multithreaded processing system, Wang et al. could process at least 25 frames per second (fps) to localize polyps during clinical colonoscopies [25]. Byrne et al. developed an AI model to differentiate diminutive adenomas from hyperplastic polyps, with a frame-processing time of 50 ms [57]. Our CAD system could achieve 50 fps for frame selection. Once the frame was selected for T/B calculation, additional time was needed for fluorescence target segmentation. The whole pipeline, including both frame selection and target segmentation, dropped the speed to ~15 fps. A real-time assistant software should operate at a speed of at least 25 fps according to PAL and NTSC standards, where video encodings are standardized to 25 or 30 fps [58]. A multithreaded system could be implemented to accelerate the processing speed. Each thread processed one image at a time. To further accelerate the speed, the input image could be down-sampled. The models with input images of 224 × 224 pixels could achieve an average of ~16 ms for suspicious frame selection and ~26 ms for fluorescence target segmentation, which decreased the processing time by 40%. We believe that future advances in computer hardware and deep-learning architecture can allow for the use of even larger input images while preserving real-time capabilities.

Several training techniques were investigated to improve the model performance. Weights pretrained on the ImageNet data corpus were used as initialization for the backbone architectures, which improved the performance for the frame selection models but not for the fluorescence target models. The input images for the fluorescence target models contained only a fluorescence channel, while the input images for the frame selection models contained both fluorescence and reflectance channels. Weights transferred from ImageNet could be applied to the reflectance channel but not to the fluorescence channel. Different augmentation techniques were tested, including (1) geometric distortions (2) brightness and contrast adjustments, and (3) image blur and noise. Looking at Table 1 and Table 2, there was no common combination of augmentations that was optimal for both datasets and tasks. Only models with geometric distortion augmentation during training demonstrated significantly improved performances in both suspicious frame selection and fluorescence target segmentation for both datasets. These geometric distortions overcame the positional and dimensional biases. On the contrary, (a) adjusting the brightness and contrast of the image and (b) adding noise or blur to the image during training showed negligible improvement in performance. However, we did not perform a search for augmentation hyperparameters. Further optimizations may further improve model performance.

Single-channel fluorescence images containing informative molecular expressions that were endoscopically invisible with conventional reflectance imaging were used in our segmentation. However, full-color images do contain important supplementary structural information on tissues and tumors that can contribute to computer-aided diagnosis and tumor segmentation. In our future work, full-color and single fluorescence channels are coregistered during imaging with a multimodal scanning fiber endoscope (mmSFE) [59] and generate four-channel 30 Hz videos with the same field of view of a 70–100 degree cone angle, which produces varying spatial resolutions across the depth of focus of 3 to 50 mm (15 microns to submillimeters, respectively) [60]. Due to the extremely high sensitivity of photomultiplier light detection on each of the four channels, the total laser power can be kept below 5 mW to reduce laser safety requirements for the clinical staff.

Adapting state-of-the-art segmentation networks from three-channel to four-channel input is straightforward by changing the convolution kernel size, but we could not directly take advantage of transfer learning from large, natural datasets, which increase the performance when the target dataset is small [61]. Applying transfer learning from an RGB dataset to a multichannel dataset is not trivial in the sense that additional channels cannot benefit from the same training as the RGB channels if no large, annotated dataset with the same additional channels exists [62]. In addition, target lesions from different modalities can have different characteristics that require feature fusion for more accurate segmentation. Working with multichannel data has been studied, and different ways of merging features have been proposed for natural images [63,64]. Segmentation using multimodality consisting of fusing multiple pieces of information to improve segmentation in medical fields is also under exploration, where fusing multiple modalities is the key challenge. According to the fusion strategies, network architectures can be categorized into input-level (feature level) fusion networks, layer-level (classifier level) fusion networks, and decision-level fusion networks [65]. Input-level fusion networks are adopted by most existing multimodal medical image segmentation networks. In this case, multimodality images are directly integrated in the input space to learn a unified feature representation, which contains the intrinsic, multimodal representation of the data [66,67]. It is used to support the learning of a traditional segmentation network. In a layer-level fusion network, images of each modality are used to learn separate feature sets of their own. These single-modality features are concatenated and fed to the decision layer to obtain the final segmentation results [68,69,70]. Like layer-level fusion, decision-level fusion segmentation networks use each modality image as the input of the single segmentation network, while in this case the final outputs of the individual networks are integrated to obtain the segmentation result. The single network can better exploit the unique information of the corresponding modality independently, which benefits when multimodal images have little direct complementary information in their original image spaces due to different image acquisition techniques [65]. Most of the fusion strategies in this category are based on averaging and majority voting [71,72]. Decision-level fusion was investigated to achieve a better performance on brain tumor segmentation among different fusion strategies because it could learn complex and complementary feature information from different modalities compared to input-level fusion networks [73]. To take advantage of different fusion schemes, some recent works have adopted three schemes simultaneously on the same dataset [66,74].

Despite the promising performance of our CAD system with real-time and automatic T/B ratio calculation, there were several limitations in this study. One limitation was that 4466 frames from only 35 patient videos were applied for training and testing the models, which limited the robustness of the models. However, we did not directly classify each frame as Barrett’s neoplasia or not. Instead, suspicious frames were selected for further T/B calculation, which did not require a relatively large volume of data containing neoplastic lesions with various appearances. For the segmentation task, the single foreground object class (fluorescence) and the high tolerance of the segmentation performance diminished the effect of the small dataset. In addition, we applied different techniques to further minimize the effect of the small dataset, e.g., image augmentation and ImageNet pretrained weights as initialization. Another limitation was that images used for model development were from patients with advanced BE or a history of neoplasia. A paucity of normal esophagus tissue limited the generalization of our model to a larger population. In addition, race and ethnicity could potentially introduce biases to machine-learning models [75]. Future studies should require larger and more-balanced datasets to assess model robustness against different medical centers, patient populations, and patient races and ethnicities.

## 6. Conclusions

We reported the successful development of a deep-learning-based CAD system for automatic and real-time T/B calculation. This accelerated CAD system based on T/B quantification provided similar high accuracy and faster processing speed (15 fps) compared with clinical studies using manual post-processing protocols without the use of pixel-level segmentation ground truth for training. Thus, this demonstration of a potentially more consistent analysis should be useful in clinical settings in three ways. First, it may serve as a reliable second observer to guide biopsies with the T/B ratio as a real-time indicator of lesion extent. Second, the system may be suitable for routine screening or surveillance using unsedated transnasal or capsule endoscopy, especially in under-served or under-developed areas where there is a lack of experienced clinicians. Third, it can be integrated into surgical procedure to provide real-time feedback during treatment. Further clinical studies should be conducted to determine if Barrett’s neoplasia detection rates may be increased and made more consistent with the assistance of a real-time CAD system.

## Figures and Tables

**Table 1:** Summary of suspicious frame selection results for different architectures (MobileNetV2 and Xception) and different combinations of augmentation.

Backbone	ImageNet	Rotation, Shift, Scale, Flip	Brightness, Contrast	Blur, Gaussian Noise	AUC
MobileNetV2		✔	✔	✔	0.926
	✔	✔	✔	✔	0.976
	✔	✔	✔		0.973
	✔	✔		✔	0.97
	✔	✔			0.969
	✔		✔	✔	0.887
	✔		✔		0.953
	✔			✔	0.926
	✔				0.915
Xception	✔	✔	✔	✔	0.989
	✔	✔	✔		**0.989**
	✔	✔		✔	0.986
	✔	✔			0.981
	✔		✔	✔	0.95
	✔		✔		0.948
	✔			✔	0.966
	✔				0.928

**Table 2:** Summary of fluorescence target segmentation results for BiSeNet model (mIOU: mean IOU over two classes).

Model + Backbone	ImageNet	Rotation, Shift, Scale, Flip	Brightness, Contrast	Blur, Gaussian Noise	Multiplex		Dimer	
					Accuracy	mIOU	Accuracy	mIOU
BiSeNet + MobileNetV2	✔	✔	✔	✔	0.977	0.868	0.959	0.844
	✔	✔	✔		0.977	0.869	0.959	0.849
	✔	✔		✔	0.976	0.864	0.962	0.858
	✔	✔			0.973	0.848	0.958	0.846
	✔		✔	✔	0.972	0.839	0.954	0.824
	✔		✔		0.97	0.828	0.952	0.824
	✔			✔	0.976	0.864	0.956	0.832
	✔				0.967	0.818	0.952	0.818
		✔	✔	✔	0.977	0.871	0.957	0.841
		✔	✔		0.977	0.87	0.959	0.846
		✔		✔	0.978	0.874	0.964	0.865
		✔			0.977	0.872	0.962	0.858
			✔	✔	0.968	0.819	0.948	0.805
			✔		0.968	0.826	0.955	0.831
				✔	0.971	0.836	0.952	0.817
					0.969	0.828	0.954	0.829
BiSeNet + Xception	✔	✔	✔	✔	0.978	0.873	0.964	0.864
	✔	✔	✔		0.978	0.874	0.962	0.859
	✔	✔		✔	0.978	0.878	0.966	0.873
	✔	✔			**0.98**	**0.886**	**0.965**	**0.868**
	✔		✔	✔	0.973	0.841	0.953	0.821
	✔		✔		0.974	0.85	0.954	0.829
	✔			✔	0.975	0.856	0.957	0.837
	✔				0.975	0.859	0.955	0.831
		✔	✔	✔	0.978	0.873	0.963	0.861
		✔	✔		0.978	0.875	0.962	0.859
		✔		✔	0.98	0.885	0.964	0.865
		✔			0.979	0.88	0.965	0.87
			✔	✔	0.97	0.827	0.956	0.83
			✔		0.971	0.833	0.951	0.815
				✔	0.972	0.84	0.956	0.834
					0.972	0.845	0.956	0.833

**Table 3:** Summary of fluorescence target segmentation results for UNet model.

Model + Backbone	ImageNet	Rotation, Shift, Scale, Flip	Brightness, Contrast	Blur, Gaussian Noise	Multiplex		Dimer	
					Accuracy	mIOU	Accuracy	mIOU
UNet + MobileNetV2	✔	✔	✔	✔	0.98	0.889	0.962	0.857
	✔	✔	✔		0.982	0.897	0.962	0.862
	✔	✔		✔	**0.981**	**0.891**	**0.968**	**0.88**
	✔	✔			0.979	0.881	0.963	0.863
	✔		✔	✔	0.974	0.847	0.955	0.829
	✔		✔		0.973	0.845	0.955	0.83
	✔			✔	0.976	0.865	0.959	0.845
	✔				0.97	0.829	0.952	0.816
		✔	✔	✔	0.979	0.88	0.963	0.865
		✔	✔		0.979	0.877	0.963	0.86
		✔		✔	0.979	0.882	0.967	0.877
		✔			0.979	0.881	0.964	0.865
			✔	✔	0.972	0.839	0.949	0.81
			✔		0.97	0.834	0.962	0.857
				✔	0.972	0.84	0.958	0.844
					0.96	0.797	0.957	0.839
UNet + Xception	✔	✔	✔	✔	0.982	0.897	0.966	0.872
	✔	✔	✔		0.979	0.883	0.965	0.87
	✔	✔		✔	0.981	0.892	0.966	0.875
	✔	✔			0.978	0.877	0.964	0.867
	✔		✔	✔	0.967	0.823	0.954	0.83
	✔		✔		0.972	0.84	0.957	0.838
	✔			✔	0.976	0.862	0.957	0.838
	✔				0.972	0.845	0.956	0.835
		✔	✔	✔	0.981	0.892	0.964	0.866
		✔	✔		0.981	0.893	0.966	0.875
		✔		✔	0.981	0.895	0.966	0.875
		✔			0.981	0.891	0.966	0.875
			✔	✔	0.969	0.831	0.96	0.852
			✔		0.971	0.841	0.958	0.844
				✔	0.973	0.851	0.96	0.85
					0.972	0.842	0.957	0.837

Note: mIOU is the mean IOU over two classes.

## Data Availability

The data presented in this study are available on request from the corresponding author. All data are not publicly available due to human testing restrictions.
